# Controlled semantic cognition relies upon dynamic and flexible interactions between the executive ‘semantic control’ and hub-and-spoke ‘semantic representation’ systems

**DOI:** 10.1016/j.cortex.2018.02.018

**Published:** 2018-06

**Authors:** Rocco Chiou, Gina F. Humphreys, JeYoung Jung, Matthew A. Lambon Ralph

**Affiliations:** Neuroscience and Aphasia Research Unit (NARU), Division of Neuroscience and Experimental Psychology, School of Biological Sciences, University of Manchester, UK

**Keywords:** Inferior frontal gyrus, Anterior temporal lobe, Semantic cognition, Connectivity, Hub-and-spoke

## Abstract

Built upon a wealth of neuroimaging, neurostimulation, and neuropsychology data, a recent proposal set forth a framework termed *controlled semantic cognition* (CSC) to account for how the brain underpins the ability to flexibly use semantic knowledge (Lambon Ralph et al., 2017; *Nature Reviews Neuroscience*). In CSC, the ‘semantic control’ system, underpinned predominantly by the prefrontal cortex, dynamically monitors and modulates the ‘semantic representation’ system that consists of a ‘hub’ (anterior temporal lobe, ATL) and multiple ‘spokes’ (modality-specific areas). CSC predicts that unfamiliar and exacting semantic tasks should intensify communication between the ‘control’ and ‘representation’ systems, relative to familiar and less taxing tasks. In the present study, we used functional magnetic resonance imaging (fMRI) to test this hypothesis. Participants paired unrelated concepts by canonical colours (a less accustomed task – e.g., pairing ketchup with fire-extinguishers due to both being red) or paired well-related concepts by semantic relationship (a typical task – e.g., ketchup is related to mustard). We found the ‘control’ system was more engaged by atypical than typical pairing. While both tasks activated the ATL ‘hub’, colour pairing additionally involved occipitotemporal ‘spoke’ regions abutting areas of hue perception. Furthermore, we uncovered a gradient along the ventral temporal cortex, transitioning from the caudal ‘spoke’ zones preferring canonical colour processing to the rostral ‘hub’ zones preferring semantic relationship. Functional connectivity also differed between the tasks: Compared with semantic pairing, colour pairing relied more upon the inferior frontal gyrus, a key node of the control system, driving enhanced connectivity with occipitotemporal ‘spoke’. Together, our findings characterise the interaction within the neural architecture of semantic cognition – the control system dynamically heightens its connectivity with relevant components of the representation system, in response to different semantic contents and difficulty levels.

## 1 Introduction

Based on an abundance of data from patients and healthy individuals, investigated using behavioural assessments, neuroimaging, and brain stimulation, Lambon Ralph, Jefferies, Patterson, & Rogers (2017) enunciated a detailed framework termed controlled semantic cognition (CSC). In CSC, representations of semantic knowledge are underpinned by a distributed system that involves *both* a pan-modality hub *and* multiple modality-specific spokes. In addition to semantic representations, there is a ‘semantic control’ system that manages how the hub and functionally diverse spokes split division of labour. Neuroimaging data have identified key regions of CSC: Performing semantic tasks activates polymodal regions generally believed to be the hub, such as the ventrolateral parts of anterior temporal lobe (ATL), as well as regions supporting executive control, such as the inferior frontal gyrus (IFG) and posterior temporoparietal regions (Binder et al., 2009, Lambon Ralph et al., 2017). Furthermore, the CSC framework makes specific predictions that, when the usage of semantic knowledge is customary and well-practised, the semantic representation system needs little input from the ‘control’ mechanisms to output a correct response (e.g., associating ducklings with eggs); by contrast, input from the ‘control’ system would ramp up when a context accentuates atypical usage of semantic information (e.g., associating ducklings with dandelion due to both being yellow) or when precise scrutiny of semantic attributes is necessary. At the neural level, it remains to be tested how different components of CSC join forces dynamically for different tasks. The principal target of this study was, therefore, to understand the flexible division of labour and functional connectivity among the hub, spoke, and executive areas in semantic cognition.

Investigations into the white-matter connectivity of the temporal lobe have identified the anatomical ‘infrastructures’ underpinning communication between the semantic control and representation systems. Using probabilistic tractography, Binney, Parker, and Lambon Ralph (2012) found that convergence of sensory information in the temporal lobe is a graded process occurring along both its coronal and sagittal axes. Along the coronal axis, different gyri of the temporal lobe connect laterally to each other, making the inferior temporal gyrus (ITG) the midpoint that receives information both from ventromedial and dorsolateral sources. Along the sagittal axis, both short- and long-range longitudinal tracts course through the temporal lobe, resulting in increasing information convergence towards the rostral temporal areas (as also found in non-human primates: Moran, Mufson, & Mesulam, 1987). In addition to this graded connectivity structure *within* the temporal lobe, white matter tracts also extend to regions *outside* the temporal lobe, such as prefrontal and parietal regions that are crucial for executive function (Fedorenko, Duncan, & Kanwisher, 2013). This includes the uncinate fasciculus that links the prefrontal cortex (particularly *pars orbitalis*) to the ATL, as well as the inferior fronto-occipital fasciculus that links the prefrontal cortex (particularly *pars opercularis* and *triangularis*) to ventral occipitotemporal areas (Bajada et al., 2017, Bajada et al., 2015). Such structural-anatomical findings align with the workflow that CSC proposes to explain how semantic cognition is implemented by the brain (Lambon Ralph et al., 2017): information from modality-specific areas (spokes) merge at the ATL (hub), where polymodal, generalisable semantic concepts are crafted by melding componential information coded in unimodal spoke areas (Lambon Ralph et al., 2010, Rogers et al., 2004). There are also abundant neural tracts linking this hub-and-spokes structure to the IFG, permitting the prefrontal system to regulate. It is crucial to note that, although the tractography evidence shows the physiological ‘hardware’ that semantic processing rests upon, it does not explain how information processing is conveyed in this structure and how it is modulated by tasks.

Our primary goal was to understand the flexible interplay between hub, spoke and executive regions under different contexts. We narrowed down this broad aim to test a specific prediction of CSC that a less-practised, atypical context that requires precise scrutiny of semantic attributes would elicit greater prefrontal-executive regulation to the hub-and-spoke system, compared to a well-practised, familiar context. This was achieved by first characterising the potential ‘hub’ and ‘spoke’ sections in the ventral temporal cortex (VTC), using a novel approach termed vectors of region-of-interest (Konkle & Caramazza, 2013) which mapped the evolution of functional responses along the VTC. We next utilised psycho–physiological interaction analyses (PPI; Friston et al., 1997) and dynamic causal modelling (DCM; Friston et al., 2003, Stephan et al., 2010) to explore how the communication between the prefrontal ‘control’ region and ‘hub-and-spoke’ structure varied between tasks. A secondary goal was to investigate how visual cortices respond to sensory stimulation (visually-presented colour patches) versus conceptual simulation of visual knowledge (colour-related concepts). We identified the occipitotemporal clusters that exhibited enhanced connectivity with the IFG (connectivity-defined clusters) and tested whether these connectivity-based clusters overlapped with those activated by perceiving sensory hue or retrieving colour knowledge. This was achieved by conducting a novel analysis of receiver operating characteristic (ROC) on the spatial distribution of sensory-, concept-, and connectivity-demarcated occipital voxels. All results were then integrated, enabling us to examine how they fit with CSC.

## 2 Materials and methods

### 2.1 Participants

Eighteen right-handed, native English-speaking volunteers (14 females, 23 ± 3 years) gave informed consent before participating in the study. All had normal colour vision, assessed using a colour-blindness test (Ishihara, 1960), completed Magnetic resonance imaging safety screening before participation, and had no neurological or psychiatric conditions. This study was reviewed and approved by the local research ethics committee.

### 2.2 Experimental design

Participants completed five functional scans. In Scans 1–3 (main tasks), they did *(i)* a colour knowledge task that required pairing semantically unrelated objects based on canonical colour, *(ii)* a semantic-associative that required pairing items based on conceptual relationship, *(iii)* a non-conceptual control task of comparing visual configurations. The contrast of colour versus semantic task allowed us to examine whether task contexts alter the interaction between the control and representational systems, as CSC would envisage.

In the colour knowledge task, participants saw a triad of words in each trial, one above the centre and the other two below, equidistant from the midline. The three words were *not* related to one another; each word referred to an object that was associated with a canonical colour (e.g., *duckling*, *dandelion*, and *plum*). Participants judged which one of the two bottom objects has a typical colour more similar to that of the top object. In the semantic knowledge task, participants also saw a triad of words (e.g., *ostrich*, *eggshell*, and *cheese*). They judged which of the two bottom words was semantically more associated with the top word. In the non-conceptual condition, we used a well-established control task (Visser et al., 2012, Visser et al., 2011) in which participants saw a triad of scrambled visual patterns. They judged which one of the two bottom patterns was the left-right mirror inverse of the top item.

We tested two different modes of semantic operation – while keeping the association strength of probe (the top word) with foil (the non-target bottom word) identical in both tasks, linking a target to a probe item in the colour task is a semantically arbitrary process that requires deliberately pairing of two words that bear minimal prior relationship, whereas linking a target to a probe in the semantic task is based on pre-existing and well-learnt knowledge. Thus, the two tasks differ on the automaticity of semantic association. To achieve this, we used the same probes and foils for the colour and semantic tasks, while they differed in the target item. For example, a ‘probe-target-foil’ triad in the colour task was ‘*mustard*, *smiley*, *hawk*’ and its counterpart in the semantic task was ‘*mustard*, *ketchup*, *hawk*’. With careful selection we ensured that *(i)* in the colour task, both the target and foil were semantically *un*related to the probe while all three words were strongly associated with a typical colour; and *(ii)* in the semantic task, neither of the two option words were associated with a colour similar to the probe's colour and only the target was semantically related to the probe. Our selection of stimuli was facilitated using latent semantic analysis (LSA), a computational technique that calculates the associative strength between word meanings from a large text corpora (Hoffman et al., 2011, Landauer and Dumais, 1997). While selecting words for each triad of stimuli, we used LSA to extract the pairwise similarity of ‘probe and colour target’ (e.g., duckling – dandelion), ‘probe and foil’ (e.g., duckling – plum), and ‘probe and semantic target’ (e.g., duckling – goose). With these corpus statistics, we examined (*i*) whether the semantic targets were more associated with probes compared to foils and colour targets, and (*ii*) whether foils and colour targets did not differ in associative strength to probes. Results of these statistical tests supported the adequacy of our stimuli set: The strength of connection for ‘probes and semantic targets’ was significantly greater, compared to that of ‘probes and foils’ and ‘probes and colour targets’ (both *p*s < 10^−16^), while no difference existed for ‘probe and foil’ versus ‘probe and colour target’ (*p* > .59). In addition, we also controlled for word length (number of letters) and lexical frequency (based on the British National Corpus) to ensure no systematic difference between conditions. The lexical stimuli set are provided in Supplemental Information (SI). Taken together, with rigorous selection and control we ensured that pairing semantic targets with probes would be easier compared to pairing colour targets with probes.^1^

Stimuli were presented in a block design, controlled using E-Prime (Psychology Software Tools). A functional scan of the main tasks contained six blocks of 19 sec for each of the three tasks and six 19-sec resting periods, giving 456 sec in duration. The order in which task conditions and stimuli sets were presented was fully counterbalanced across our sample of 18 participants so that each condition and stimuli was equally likely to appear in every possible position of the sequences, with stimuli randomly drawn from a designated stimuli-set for a given scan and shuffled across blocks. A task block contained five trials. Each trial began with a fixation display prompting task demand (*colour*, *association*, or *inverse*), presented for 800 msec. Subsequently, a triad of stimuli (words or scrambled patterns) was displayed for 3 sec during which participants had to indicate their choice by pressing one of the two designated buttons on a MR-compatible response pad. All visual stimuli were black and displayed on a mid-grey background, presented via a mirror mounted on the head coil. The target options were equally likely to appear on the left and right side. Prior to entering the scanner, participants completed two practise blocks for each task.

In Scans 4–5, participants did the Farnsworth-Munsell 100 Hue Task (Hsu et al., 2011, Simmons et al., 2007) that localised brain regions underlying colour perception. In each trial participants saw an annulus stimulus presented centrally. The annulus comprised five ‘wedges’ and could be coloured or greyscale (lightness matched to its counterpart coloured stimulus; presented in separate blocks of trials). The five component wedges were varied in lightness, arranged either in an orderly sequence (from lightest to darkest; 50% of the trials) or disorderly (50%). Among the sequentially-ordered stimuli, the wedges were equally likely to move from lightest to darkest clockwise and anti-clockwise. A localiser scan contained 10 blocks of 15 sec for each of the coloured and greyscale conditions, with a 10-sec resting period between blocks, giving 500 sec in duration. The coloured and greyscale blocks were randomly interleaved within a scan and counterbalanced across participants. Each block contained 5 trials. At the beginning of a trial, a fixation display was shown (500 msec), followed by the annulus stimuli (2.5 sec). Participants were asked to judge if the lightness of component wedges was arranged sequentially or not by pressing a designated button. They performed the localiser task after they had completed the main tasks.

### 2.3 MRI acquisition

All scans were acquired using a 3T Phillips Achieva scanner equipped with a 32-channel head coil and a SENSE factor of 2.5. A dual gradient-echo EPI sequence was used to maximise signal-to-noise ratio in the ventral ATLs (Halai, Welbourne, Embleton, & Parkes, 2014). Using this technique, each scan consisted of two images acquired simultaneously with different echo times: a short echo optimised to obtain maximum signal from the ventral ATLs and a long echo optimised for whole-brain coverage. The sequence included 31 slices covering the whole brain with repetition time (TR) = 2.8 sec, short/long echo times (TE) = 12/35 msec, flip angle = 85°, field of view (FOV) = 240 × 240 mm, resolution matrix = 80 × 80, slice thickness = 4 mm (no interleaving gap), and in-plane resolution = 3 × 3 mm. To reduce ghosting artefacts in the temporal lobes, all functional scans were acquired using a tilted angle, upward 45° off the AC-PC line. Functional scans of the main tasks were collected over three runs; each run was 456-sec long during which 163 dynamic scans were acquired (alongside 2 dummy scans, discarded). Functional scans of the localiser task were collected over two runs; each run was 500-sec long during which 179 dynamic scans were acquired (alongside 2 dummy scans). To tackle field-inhomogeneity, a B0 field-map was acquired using identical parameters to the functional scans except for the following: TR = 599 msec, short/long TEs = 5.19/6.65 msec. Total B0 scan time was 1.6 min. A high-resolution T1-weighted structural scan was acquired for spatial normalisation; this included 260 slices covering the whole brain with TR = 8.4 msec, TE = 3.9 msec, flip angle = 8°, FOV = 240 × 191 mm, resolution matrix = 256 × 163, and voxel size = .9 × 1.7 × .9 mm. The structural scan took 8.19 min.

### 2.4 Pre-processing and generalised linear model (GLM) analysis

Analysis was carried out using SPM8 (Wellcome Department of Imaging Neuroscience, London). The functional images from the short and long echoes were integrated using a customised procedure of linear summation (Halai et al., 2014, Poser et al., 2006). The combined images were realigned using rigid body transformation (correction for motion-induced artefacts) and un-warped using B0 field map (correction for field-inhomogeneity). The averaged functional images were co-registered to each individual participant's T1 structural scan. Spatial normalisation into the MNI space was performed using the standardised DARTEL protocol by group-wise registration of individual's grey and white matter into a template brain created from the group average (Ashburner, 2007). This optimises inter-participant alignment, allowing more precise localisation. The functional images were then resampled to a 3 × 3 × 3 mm voxel size. Voxel-smoothing was applied using an 8-mm Gaussian FWHM kernel, in accordance with the default setting of SPM. Contrasts of interest were estimated using general linear models convolving a box-car function of all experimental conditions with a canonical haemodynamic response function (main tasks: the colour, semantic, and control conditions; localiser: the coloured and greyscale conditions), with resting periods modelled implicitly. Motion parameters and reaction times were entered into the model as parametric covariates of non-interest, which accounted for brain activities driven by head movements and task difficulty/effort. Low frequency drifts were removed using a high-pass filter of 128 sec.

### 2.5 Vectors-of-ROI analysis

**Fig. 1:**
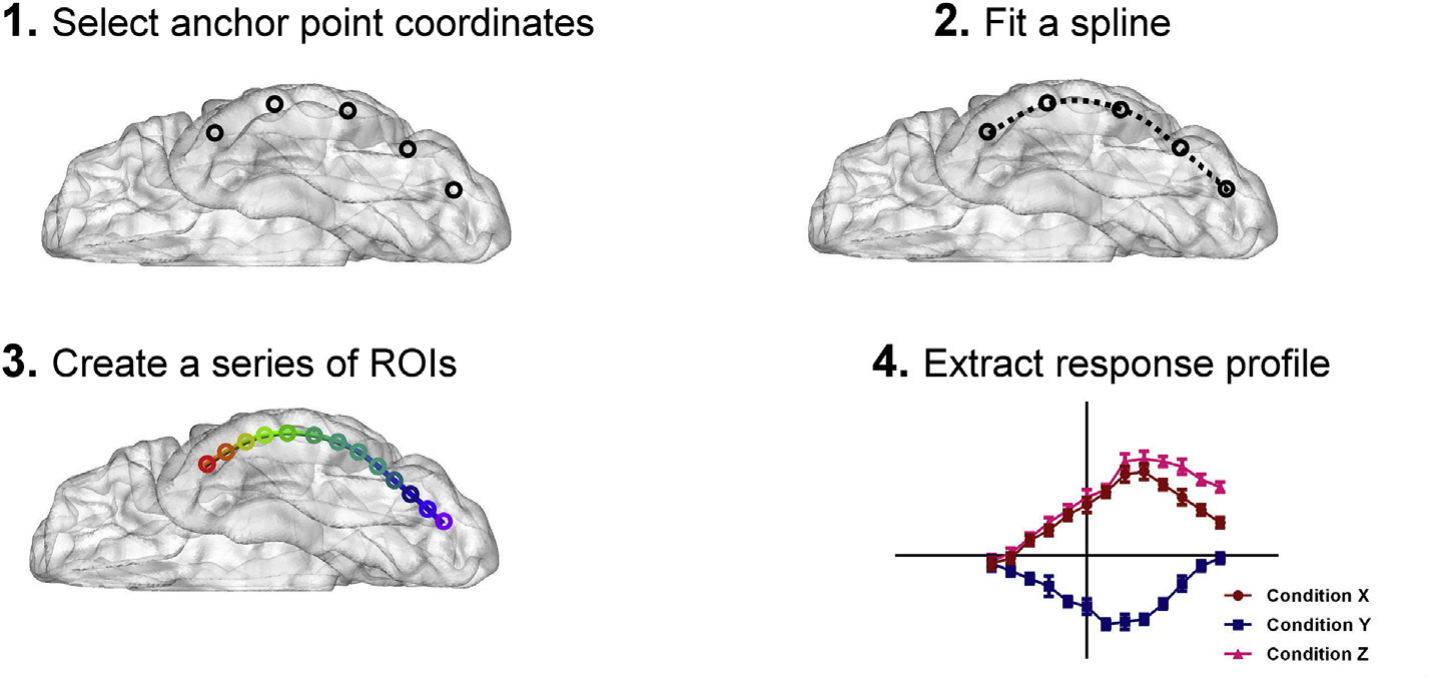
Schematic illustration for the procedure of constructing a vector of ROIs along the inferior temporal gyrus (ITG); the same procedure was performed for the fusiform gyrus. The steps contained: (1) specify a series of anchor points along a cortical path of interest (e.g., MNI coordinates along the ITG from its posterior to anterior sections), (2) fit a spline through these anchor points using piece-wise linear function, (3) define a series of evenly spaced anatomical spherical ROIs along this spline, and (4) compute the response strength (*β* weight) for all contrasts of interest (colour *vs*. control, semantic *vs*. control, colour *vs*. semantic) in each ROI.

We performed a vectors-of-ROI analysis (Konkle & Caramazza, 2013) to explore the evolution of preferential response for different tasks. As illustrated in Fig. 1, the procedure of constructing a vector contained the following steps: *(i)* Along the ventral pathway, we specified a medial and a lateral sector based on the demarcation of anatomical template, and for each sector we secured a series of anchor points on a cortical path of interest; *(ii)* we fitted a spline across these anchor points using linear approximation of piecewise function; *(iii)* we created a series of spherical ROIs, evenly distributed along the spline; *(iv)* we computed and extracted the response strength for all contrasts of interest in each ROI (*β* weights for ‘colour *vs*. control’, ‘semantic *vs*. control’, and ‘colour *vs*. semantic’). Specifically, based on the sulcal/gyral definitions given in the Wake Forest University Pickatlas toolbox (Maldjian, Laurienti, Kraft, & Burdette, 2003) we first segregated the ventral occipital and temporal cortices into two compartments: one sector included the lateral occipital complex concatenated with the inferior temporal gyrus, and the other sector comprised the lingual and fusiform gyri. Subsequently, we partitioned each sector into five segments of approximately equated length (on the *y*-axis) and set the centroid of each segment as the ‘anchor points’. Using a function of piecewise linear approximation, we fitted a ‘spline’ across the five anchor points for each sector. On each spline, the piecewise distance between each pair of anchors was divided into three equal pieces, giving two intermediate points between anchors. Finally, a series of 13 non-overlapping spherical ROIs (radius = 3 mm) was created, centred at the five anchors and eight intermediate points. The two vectors of ROI spanned from the occipital to the temporopolar cortex (range on the *y*-axis: −78 to 5). Great care was taken to adjust the coordinates so that on the coronal and axial planes the ROIs of the two vectors were matched on the *y*-axis and *z*-axis, with them only differing on the sagittal plane (*x*-axis). For each contrast of interest, we extracted *β* weights for each ROI sphere from the GLM results for further statistical analysis.

### 2.6 PPI

We investigated whole-brain functional connectivity to the left ventral ATL and IFG using PPI analyses. We first extracted the time-series of estimated neural signal from a seed region (the left ATL/IFG) and constructed a GLM model containing three factors: a psychological variable (contrast of interest), a physiological variable (seed activity), and a psychophysiological variable (the interaction term of the former two). For each participant and each run of main tasks, BOLD activity was extracted from a sphere (radius = 6 mm) centred on the local maxima of ATL and IFG activation identified by the relevant contrasts in GLM and converted into neural time-series using the standard deconvolution algorithm of SPM8. We then performed a whole-brain search to identify voxels whose activity could be explained by the PPI factor. We conducted two PPIs: *(i)* colour *vs*. semantic, with ATL seed, *(ii)* colour *vs*. semantic, with IFG seed.

### 2.7 DCM

DCM analysis was performed using DCM10 included in SPM8. As the considerations for constructing the DCM were based on the results of other analyses, to improve comprehensibility we report the details about rationales, model configuration, and selection of nodes in the Results section 3.6 after we have reported the results that DCM relied upon. Here we outline key information about our DCM: We tested two models; the ‘top-down’ model assumes that performing atypical pairing based on colour knowledge would enhance modulatory signals flowing ‘downstream’ from the prefrontal cortex to colour-related clusters of ventral occipitotemporal cortex. The contrasting ‘bottom-up’ model has otherwise identical structure and nodes, but instead assumes that the colour task would reinforce feed-forward information moving from occipitotemporal to prefrontal cortex. For each participant and each of the five target nodes (the left IFG, ventromedial ATL, ventrolateral ATL, occipital ‘concept’ area, and occipital ‘percept’ area), we extracted the BOLD time-series. The target nodes were localised based on individual's maximal response closest to the peak activation point identified in group analysis and defined as spherical ROIs of 6-mm radius; BOLD series were converted into neural activity using the first eigenvector extracted by the default algorithm of SPM8. For both the ‘top-down’ and ‘bottom-up’ model, the five nodes were all set to be bi-directionally connected with one another. Each model tests 20 ‘endogenous’ parameters that reflect baseline connectivity in the absence of experimental perturbation, as well as five parameters that reflect changes due to different experimental contexts. Subsequently, we examined the explanatory power of each model by performing fixed-effect (FFX) Bayesian selection, comparing the top-down model against the bottom-up model, using the algorithm implemented in SPM8 (Stephan, Penny, Daunizeau, Moran, & Friston, 2009). The FFX analysis assumes that the optimal model would be identical across individuals. It computes ‘*model posterior probabilities*’, which gauge each model's ability to interpret the causative strength of synaptic connections among neuronal populations (nodes) and their susceptibility to contextual modulation. This allowed us to assess the probability that one model offers a better description for a given data-set than another model. However, because the FFX analysis might be vulnerable to outliers, we also implemented a random effect (RFX) analysis, which took into consideration the heterogeneity of model structure across individuals. It uses hierarchical Bayesian modelling that calculates parameters of a Dirichlet distribution which describes the probabilities of both models considered. These probabilities define a multinomial distribution over model space, enabling the computation of the posterior probability (the likelihood of being true) of each model given the data of all subjects and the models considered. The results of RFX analysis are reported in terms of ‘*model exceedance probabilities*’, indicating if one model is more probable to hold true than the other model.

### 2.8 Receiver operating characteristic (ROC) analysis

The ROC analysis was performed based on the results of the initial whole-brain GLM and PPI analyses. The rationales and procedures of the ROC analysis are described in detail in Results section 3.7. Here we only provide key information: We first identified all active voxels showing sensitivity to colour perception (localiser: coloured > greyscale), colour concept (main tasks: colour > semantic), and colour-related co-variation with the IFG (PPI: colour > semantic) in the left occipital lobe mask, threshold at *p* < .01. This liberal threshold was purposefully used to allow scrutiny of the full breadth of different activity levels, from weakly to strongly responsive. All identified voxels were ranked based on their *t* value (activation strength) into a percentile. We then examined the location of each voxel, starting from the most active voxel to the least. For each type of colour processing (concept, percept, and PPI), three ROC curves for each functionally-defined target ROIs (concept-specific, percept-specific, and PPI) were created. The computation of each ROC function began with the voxel most active for a certain effect, and finished with the least active. After constructing the ROCs, we calculated values of area under the curve (AUC) to quantify the likelihood of voxels appearing in a certain region.

## 3 Results

### 3.1 Behavioural data

Accuracy was high for the three main tasks. As expected, there was a main effect of task (*F*_2, 34_ = 12.27, *p* < .001, *η*_p_^2^ = .42), with arbitrary pairing by canonical colour (86%) being less accurate than semantic pairing by meaning (94%) and the control task (91%). This effect was also evident in RTs (*F*_2, 34_ = 16.70, *p* < .001, *η*_p_^2^ = .49), with slower RTs for the colour (1569 msec) than semantic (1387 msec) and control (1354 msec) tasks. These results are consistent with our prediction that arbitrary pairing would be more effortful than the other tasks. In the localiser experiment, accuracy and RTs did not significantly differ between coloured (83%; 1123 msec) and greyscale (82%; 1144 msec) stimuli (both *p*s > .24). Note that in subsequent fMRI analysis, a parametric modulator of reaction time was included to account for and rule out its potential influences.

### 3.2 Whole-brain analysis

**Fig. 2:**
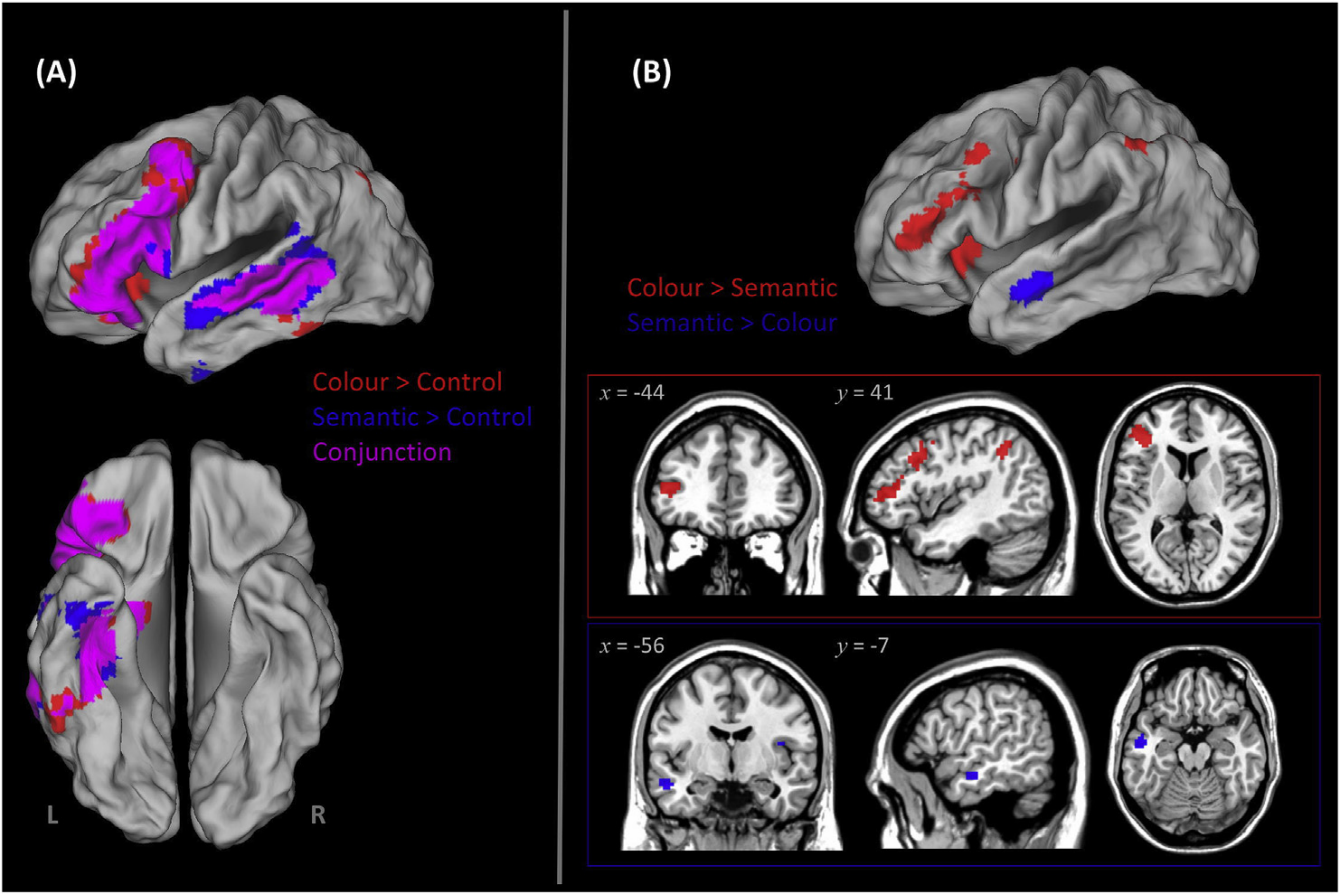
Results of whole-brain analysis, stringently thresholded at *p* < .05 (family-wise error corrected for multiple comparisons) for clusters and *p* < .0001 for voxel intensity. **(A)** Regions showing significantly greater activation for arbitrary pairing by canonical colour than control (red), semantic pairing by usual meaning than control (blue), and conjunctions of the two contrasts (magenta). **(B)** Clusters showing significantly greater activation for colour pairing than semantic pairing (red) and the reverse contrast (blue). Shown in the inset boxes are left frontoparietal clusters (the ‘control’ module) more active for colour pairing (red) and left anterolateral temporal cluster sensitive to semantic pairing (blue).

The whole-brain interrogation was stringently thresholded at *p* < .0001 for voxel intensity and *p* < .05 (FWE-corrected for multiple comparisons) for clusters. Relative to the control task, both colour and semantic decisions elicited greater activation in a strongly left-lateralised distributed network well-established in previous inquiries of semantic memory, including the IFG and a vast swathe of the temporal cortex, encompassing superior to inferior subregions (see Fig. 2). Critically, both colour and semantic tasks triggered robust activation in the ATL (the anterior fusiform gyrus, FG), an area reliably identified as the key substrate of semantic ‘hub’^2^ (Binney et al., 2010, Mion et al., 2010, Shimotake et al., 2014, Visser et al., 2010). Compared to semantic pairing (colour > semantic), colour pairing enhanced activation in the prefrontal ‘executive control’ regions, including the IFG and its neighbouring middle frontal gyrus, as well as the intraparietal sulcus. This dovetails with the prediction of CSC that tasks demanding atypical and precise processing necessitate more input from the ‘control’ network, hence heightened activity in the frontoparietal network. The reverse contrast (semantic > colour) revealed greater activation in the dorsolateral aspect of the ATL.

### 3.3 Colour concept *vs*. colour percept

**Fig. 3:**
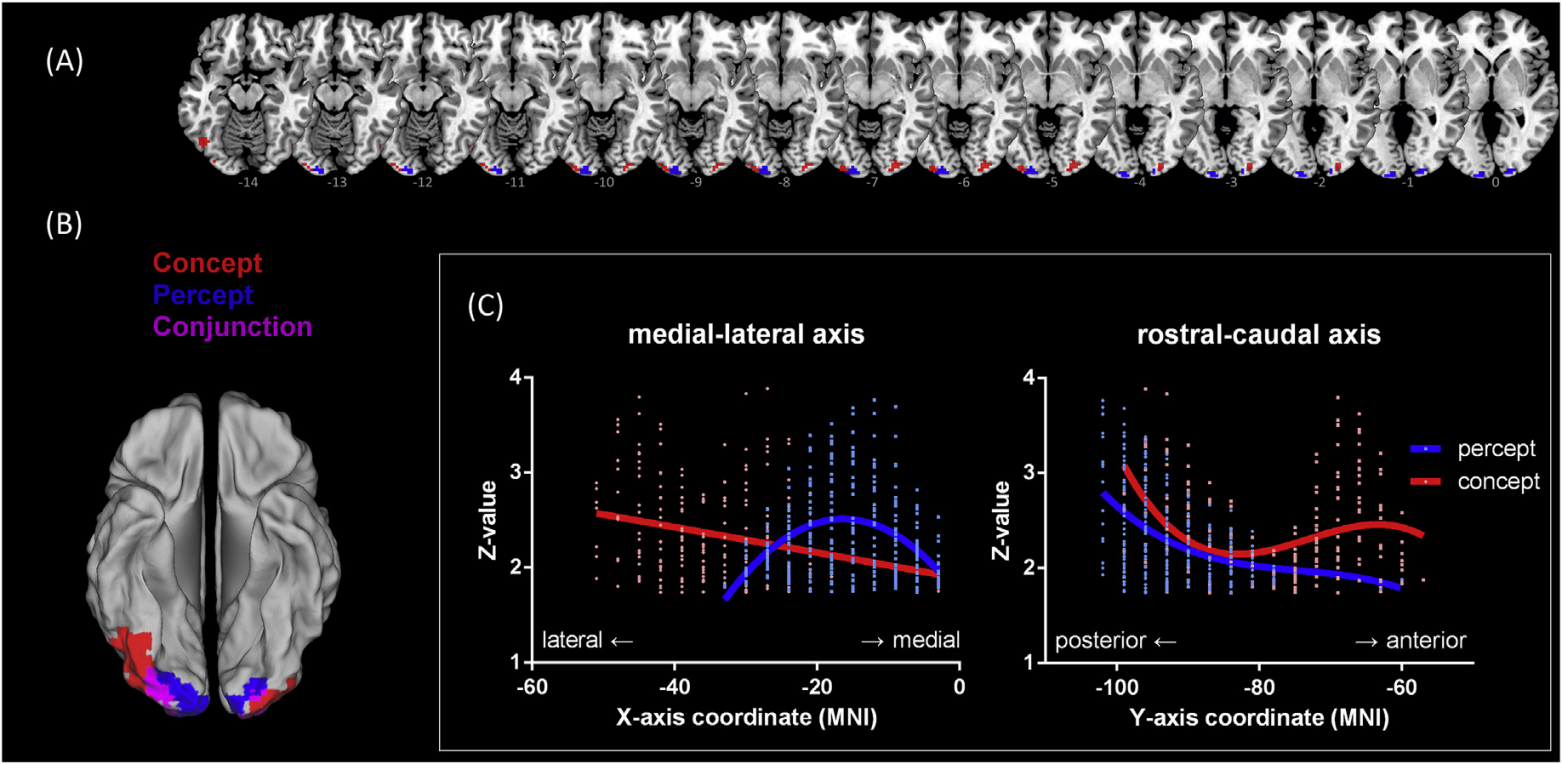
(A) Views of axial slices displaying percept *vs*. concept voxels, ranging from *z* = −14 to 0 (MNI). The occipitotemporal regions significantly more active for colour percept are shown in blue (localiser task: coloured > greyscale), more active for colour concept are show in red (main tasks: colour pairing > semantic pairing), and their overlaps are in magenta. Statistics are thresholded at *p* < .005 for voxel intensity and further constrained at least 270 mm^3^ for cluster size. **(B)** For illustration purposes only to show the cortical layout of ‘percept *vs*. concept’ areas, active clusters are rendered on an inflated template, thresholded at *p* < .05. **(C)** Mean-corrected activation level (*Z*-statistics) of occipital voxels as a functional of triggering stimuli (percept, concept) and location along the *x*- and *y*-axis, with the polynomial regression best-fitting lines representing trends.

The ‘hub-and-spokes’ view postulates that concepts, particularly those about perceptual features, engage the ATL hub *plus* modality-specific ‘spoke’ cortices. Thus, we specifically focused on the visual cortices, examining the extent to which colour knowledge involved the visual regions and comparing it to colour perception. We independently identified occipital voxels sensitive to colour percept (chromatic > greyscale, localiser task) and colour concept (colour > semantic, main task), thresholded at *p* < .005 for each voxel and further limited by a cluster constraint of contiguously extending at least 270 mm^3^. Consistent with the ‘hub-and-spoke’ view and embodied cognition (for review, see Barsalou, 2008, Martin, 2015), we found percept and concept engage adjacent sections of the visual cortex (Fig. 3A). Interestingly, while percept and concept voxels adjoin and partially overlap, we noticed that percept tends to be situated more medially and posteriorly whereas concept tends to be located more laterally and anteriorly. This separation is more manifest when rendered on the cortical surface with a liberal threshold (Fig. 3B). To systematically delineate the spatial layout of percept *vs*. concept voxels, we extracted the normalised activation strength (*Z*-value) of left-hemisphere voxels as a functional of position and represented content, with best-fitting polynomial functions representing the trend of this clearly separable cortical distribution. As illustrated in Fig. 3C, percept and concept voxels were distributed in a *graded* fashion, with a lateral and anterior shift into conceptual processing. This is consistent with a representational gradient, arguing against clear-cut modular separation. It is noteworthy that, while we were able to detect the effect of colour knowledge > semantic association in broad expanses of prefrontal ‘control’ areas with stringent criteria (voxel: *p* < .0001, cluster: *p* < .05 FWE-corrected; see Fig. 2), we had to apply liberal thresholds^3^ to detect the same effect in the visual cortex (voxel: *p* < .005, cluster: at least 270 mm^3^). This points to a need to recognise that whilst modality-specific embodied representations contribute as building blocks for concepts (especially during the initial acquisition of a novel concept), they have to be considered together with higher-level regions as constituents of a greater functional neural architecture.

### 3.4 Vector-of-ROI analysis: the evolution of representational preferences

**Fig. 4:**
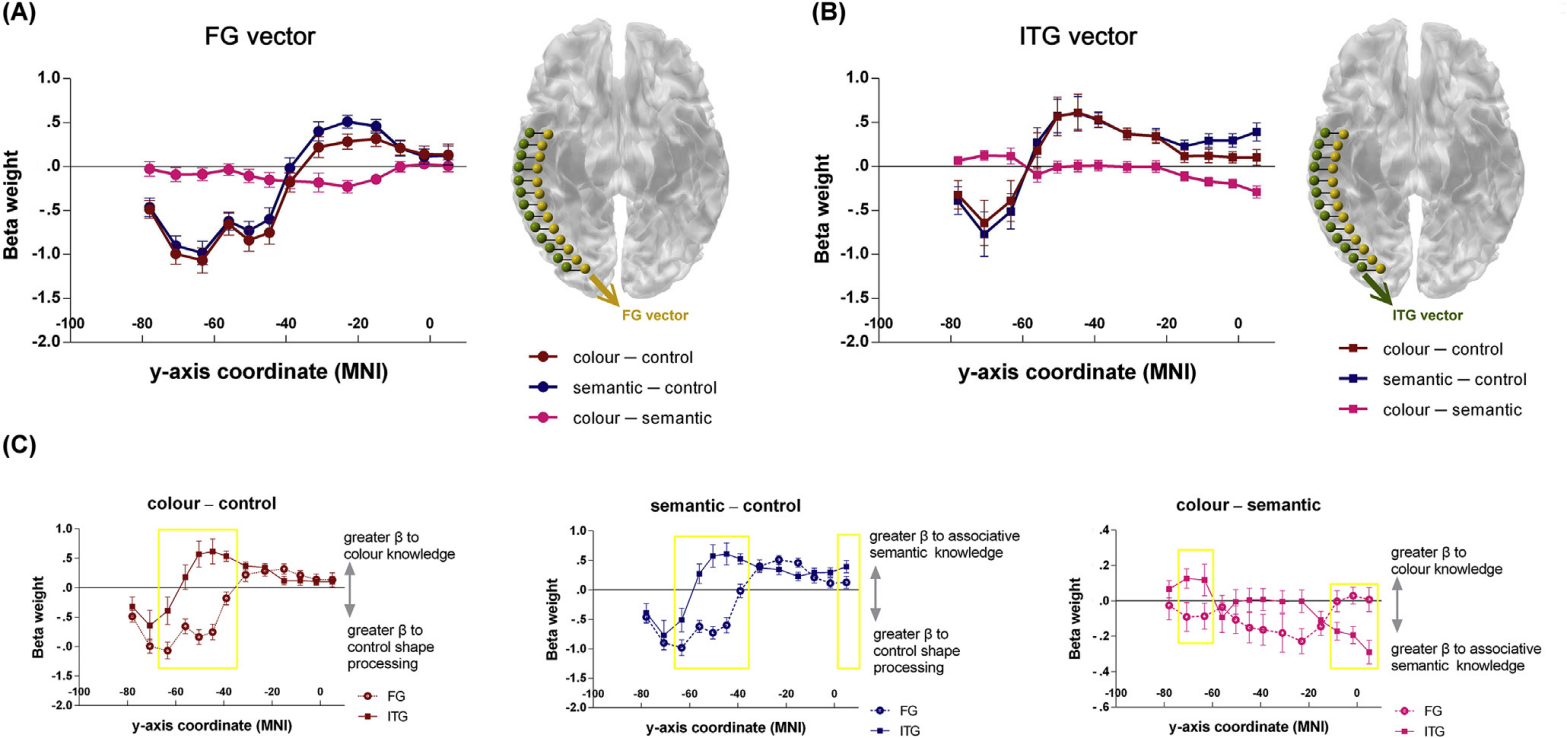
**(A)** Beta weights for each contrast of interest (red: colour pairing *vs*. control, blue: semantic pairing *vs*. control, magenta: colour pairing *vs*. semantic pairing) as a function of position along the *y*-axis of the FG vector. **(B)** Beta for each contrast of interest as a function of position along the ITG vector. Error bars reflect ±1 SEM. Note that the control task was visual decisions processing scramble patterns. **(C, left)** Estimates of activation (Beta weights) for the ‘colour pairing > control’ contrast as a function of vectors of ROI (FG *vs*. ITG) and location along the *y*-axis. **(C, middle)** Beta weights for the ‘semantic pairing > control’ contrast as a function of vectors and location. **(C, right)** Beta weights for the ‘colour pairing > semantic pairing’ contrast as a function of vectors and location. Golden boxes mean statistically significant differences between the FG and ITG vectors. Note all pair-wise tests were performed based on significant interactions that had already controlled family-wise error of multiple comparisons.

To explore how neural responses unfold along the caudal ‘spoke’ and rostral ‘hub’ zones of ventral-temporal pathway, we performed a vectors-of-ROI procedure (Konkle & Caramazza, 2013). Our paradigm provided optimal circumstances for studying this transition: the control task demanded pairing stimuli using perceptual conformation; the semantic task demanded pairing concepts using meaningful relationship; whilst the colour knowledge task straddled between perception and semantics (pairing concepts using their known perceptual features stored in semantic memory). We created a series of parallel, non-overlapping spherical ROIs along the FG and ITG, spanning ventral occipital and temporal cortices (from *y* = −78 to +5), and extracted β-weight for each contrast of interest. Along both the vectors of FG (Fig. 4A) and ITG (Fig. 4B) we observed clear gradients evolving from perception to semantics. Both gyri shifted from being more active for visual configuration processing (control task) to conceptual knowledge (the colour and semantic tasks) as neural processing proceeded anteriorly. Critically, closer scrutiny of Fig. 3B and C revealed two functional distinctions between the FG and ITG vectors: *(i)* the ‘tipping point’ that switched from perceptual to conceptual processing was evidently more posterior in the ITG than FG vector; and *(ii)* the two vectors also differed in their response profiles in the most rostral poles of ROIs; anterior FG was equally responsive to the colour and semantic knowledge tasks, whereas anterior ITG was apparently more active for the semantic task than colour task.

Formal statistical analysis fully corroborated these visual inspections (Fig. 4C). We obtained a three-way interaction, indicating that the evolution of neural responses across the ROIs significantly differed between contrasts and vectors (*F*_24, 408_ = 10.03, *p* < .001, *η*_*p*_^2^ > .37). For colour *vs*. control, the difference of FG *vs*. ITG activations significantly interacted with anterior-to-posterior positions (*F*_12, 204_ = 15.21, *p* < .001, *η*_*p*_^2^ > .47; Fig. 4C left). The two vectors significantly differed in the posterior-to-middle segments (*y* = −63 to −39), with ITG regions exhibiting greater response to colour knowledge than did FG regions (all *p*s < .01, indicated by the golden rectangle). However, in more rostral and most caudal cortices, the two vectors did *not* differ in response profile (all *p*s > .1). For semantic *vs*. control, the two vectors' response also significantly interacted with positions (*F*_12, 204_ = 12.54, *p* < .001, *η*_*p*_^2^ > .42; Fig. 4C middle): Compared to the FG vector, the ITG vector displayed greater response to the semantic task in the posterior-to-middle sections (*y* = −63 to −39) and, crucially, in the most rostral ROI, situated in the temporal pole (*y* = +5; all *p*s < .05; note this rostral, temporopolar section showed no *between-*gyri difference for colour *vs*. control while exhibiting a significant difference for semantic *vs*. control). Finally, in the direct contrast of colour *vs*. semantic decisions, the difference between the two vectors was evident in the most posterior and anterior extremes (interaction: *F*_12, 204_ = 6.64, *p* < .001, *η*_*p*_^2^ > .28; Fig. 4C right): in the caudal segment (*y* = −71 to −63), the ITG was more active for the colour knowledge task, whereas in the rostral segment (*y* > −8), the ITG was more active for the semantic association task, relative to the fusiform ROIs (all *p*s < .05).

### 3.5 PPI

**Fig. 5:**
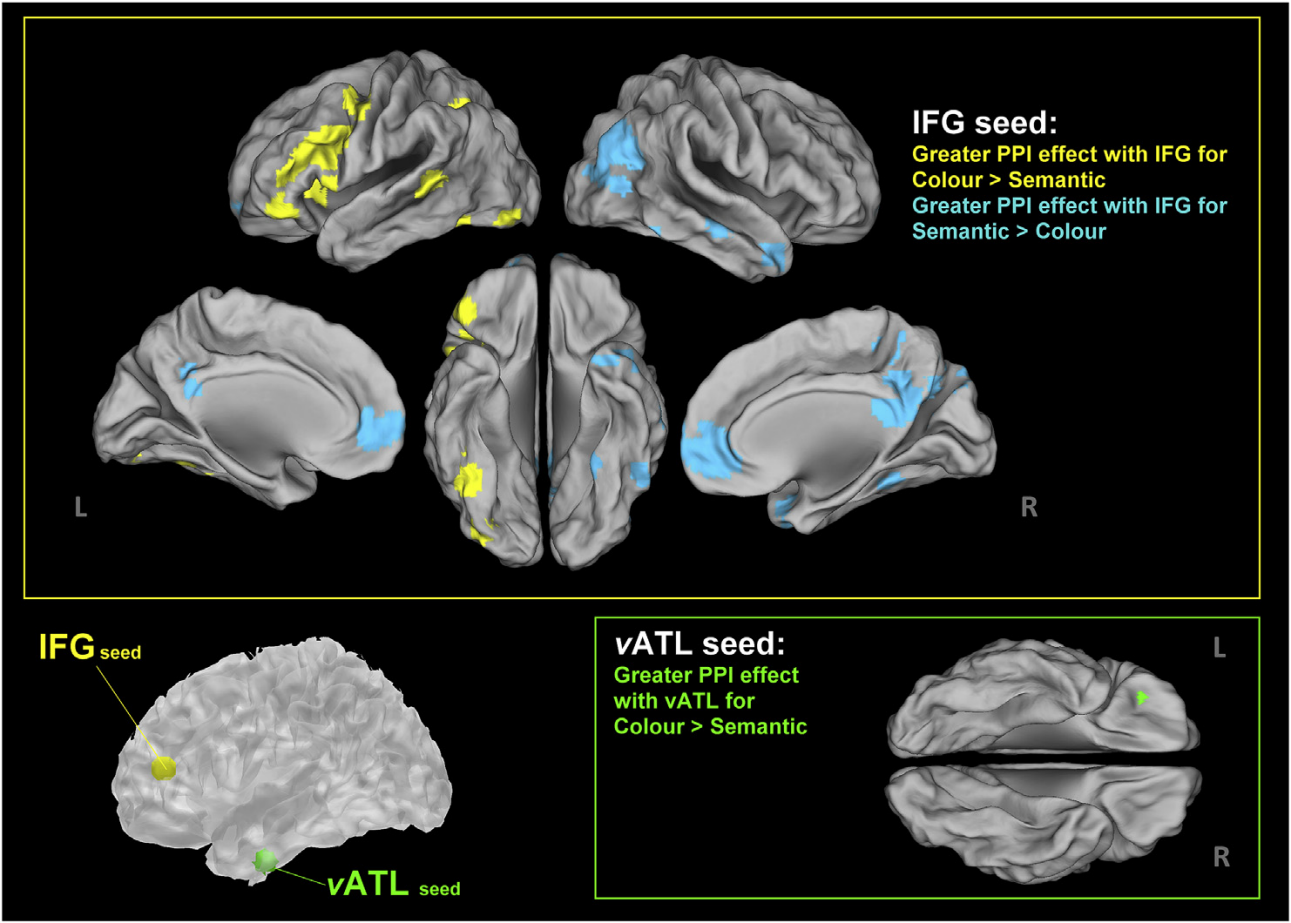
Results of the PPI analysis, thresholded at *p* < .005 for voxel intensity and furthered constrained for cluster size (extending at least 270 mm^3^). Yellow: significantly greater functional connectivity (the PPI effect) with the left IFG seed for arbitrary pairing by canonical colour. Cyan: significant PPI effect with the left IFG for semantic pairing by meaning. Green: significant PPI effect with the left ventral ATL seed for colour pairing.

To investigate how performing different tasks altered connectivity to the ‘semantic control’ and ‘semantic representation’ systems, we conducted PPI, thresholded at *p* < .005 for voxel and further constrained cluster size (contiguous extension ≥ 270 mm^3^). We know that, compared to pairing using well-learnt semantics, deliberate pairing by colour was behaviourally more effortful and elicited greater activation of the prefrontal cortex. Thus, we tested a key prediction of CSC that the more demanding colour task might augment communication between the prefrontal ‘control’ system and the ventral-temporal ‘representation’ regions, relative to the semantic task. We localised the seeds at the ATL ‘hub’ (anterior FG) and the prefrontal ‘control’ region (the IFG) using peaks of the initial GLM analyses, and compared the contextual connectivity of colour knowledge > semantic association. For the ATL seed, PPI showed that a cluster of the orbitofrontal cortex was more connected with the ‘hub’ during the colour than semantic task (Fig. 5); this orbitofrontal cluster is evidently more ventral than the IFG seed, which is situated at the dorsal part of *pars triangularis*. For the IFG seed, PPI revealed two major groups of brain regions showing enhanced connectivity to the IFG: (*i*) Widely distributed areas of ‘control’ network, including an extensive stretch of left prefrontal ‘executive-control’ regions, spreading dorsally (to the middle frontal gyrus) and ventrally (to *pars orbitalis*), as well as the intraparietal sulcus and the posterior middle/superior temporal gyrus. (*ii*) Most intriguingly, the left visual ‘spoke’ cortex was also linked to the IFG, peaking at the lateral occipital area and middle fusiform gyrus (Fig. 5). The pattern of connectivity to the IFG revealed key features of the neural architecture for colour knowledge: To access specifics of remembered colour attributes, the IFG becomes more tightly connected with other nodes of the frontoparietal ‘control’ network. Furthermore, its connectivity with the occipitotemporal ‘spoke’ cortex is also reinforced. This particular channel of connectivity might provide a crucial avenue for the prefrontal control centre to retrieve the embodied representations of colour stored in the occipitotemporal regions.

In the reverse test (‘semantic > colour’) with the IFG seed, we found various nodes of the default mode network (DMN; Anticevic et al., 2012, Spreng and Grady, 2010) were more connected to the seed, including the angular gyrus, cuneus, and medial prefrontal cortices (Fig. 5). Further analysis showed that this stronger IFG-DMN connectivity during the semantic condition was driven by greater *deactivation* of the DMN during the more demanding colour task (for details see SI), consistent with its propensity to deactivate during cognitively taxing situations (Humphreys, Hoffman, Visser, Binney, & Lambon Ralph, 2015).

### 3.6 DCM

The aim of DCM is to make directional inferences about the impact that one brain region exerts over another and how this is affected by tasks. We used DCM to adjudicate two hypotheses that could potentially explain our PPI results. The top-down hypothesis posits that the PPI connectivity reflects the prefrontal ‘control’ regions modulating the occipitotemporal ‘spoke’ regions via giving feedback signal. This might be due to imagery during the colour task (e.g., conjuring up mental pictures of object colours, a common strategy for the task). By contrast, the bottom-up view assumes that activities of the ‘spoke’ areas arise early and precede subsequent higher-level processing that leads to semantic decisions; thus, it would be the ‘spoke’ feeding forward to the ‘control’ region. We selected the nodes of DCM network based on the following empirical evidence of our prior GLM, PPI, and vectors-of-ROI analyses, and localised the nodes based on GLM outcomes.

i. Both colour knowledge and associative semantics drove robust activation of the anterior FG (the semantic ‘hub’), peaking at medial-ventral parts of the ATL, as the GLM and ROI-vectors analyses both showed;
ii. The colour knowledge task induced greater activation of the left IFG (control centre) and lateral occipitotemporal regions (modality-specific ‘spoke’) relative to associative semantics, as indicated by the GLM and ROI results;
iii. The associative semantic task induced greater activation of the anterior ITG (lateral-ventral parts of the ATL) relative to colour knowledge, as revealed by the ROI-vectors analysis (the GLM results similarly revealed the anterior ITG, when thresholded more liberally);
iv. The occipital concept-related (colour knowledge > associative semantics) clusters were adjacent to the percept-related (colour images > greyscale) clusters, with a trend shifting laterally and anteriorly for concept processing, as shown by the GLM analysis;
v. The colour knowledge task enhanced functional connectivity between the ‘control centre’ IFG and the visual ‘spoke’ clusters (the lateral occipital cortex and posterior FG), as revealed by the PPI analysis.

**Fig. 6:**
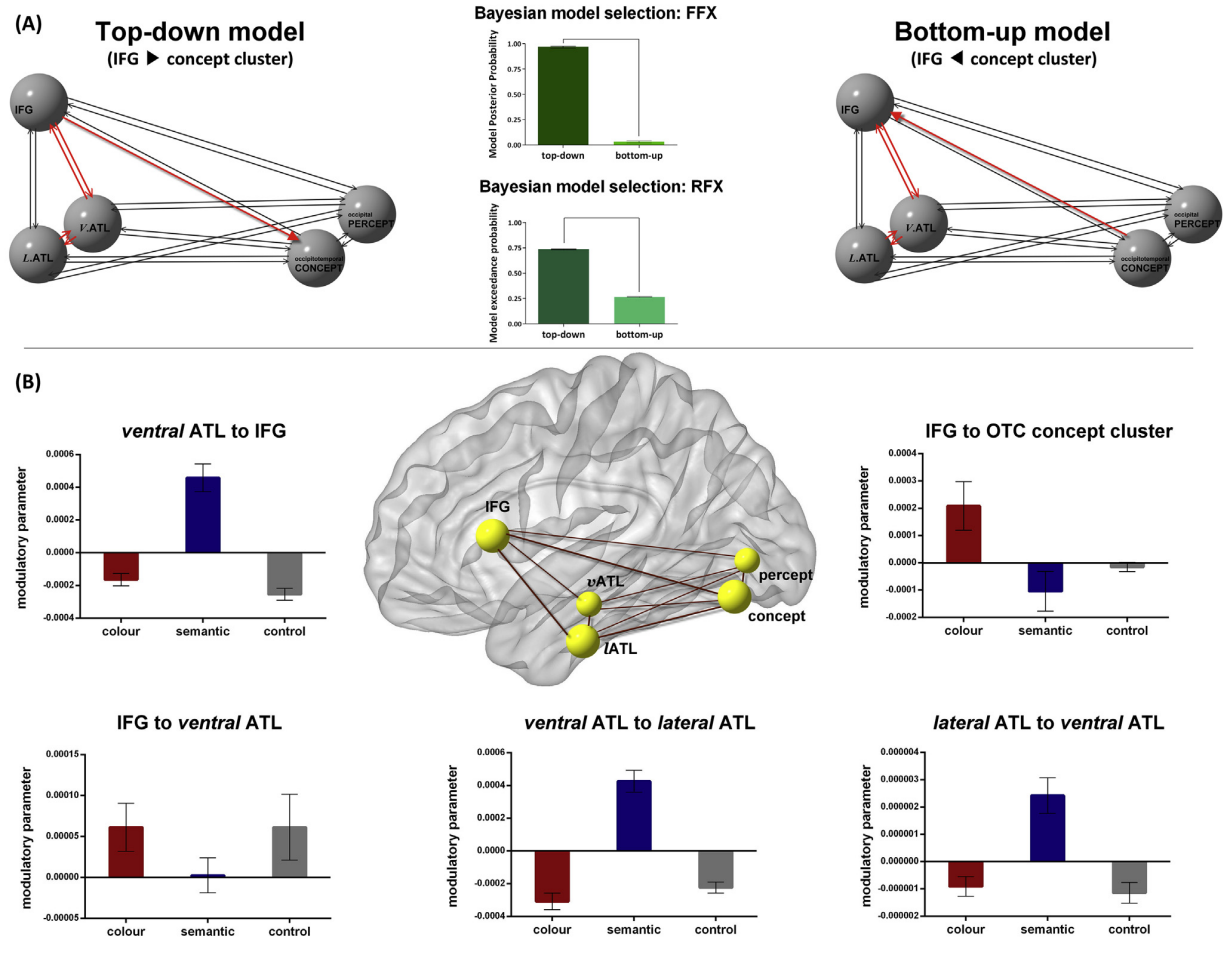
(A) Schematic representation of the models tested (left: top-down model; right: bottom-up model) and the outcome of both fixed-effect (FFX) and random-effect (RFX) Bayesian model selection (middle). Black arrows represent intrinsic ‘baseline’ connections between nodes without the modulation of task contexts. Red arrows represent the connections hypothesised to be susceptible to the impact of experimental contexts. **(B)** Statistically significant changes of causative connectivity (estimates of modulatory parameters) of the ‘winner’ top-down model, plotted for each of the five assumed context-sensitive connections.

Based on the considerations and evidence laid out above, we constructed the DCM models using the regions that showed significant effects in the relevant contrasts of interest. The models consisted of bilateral, intrinsic connections (thin black arrows) between the IFG, the *ventral* ATL (anterior FG/hub), the *lateral* ATL (anterior ITG), the lateral occipital *colour-concept* cluster, and the medial occipital *percept* cluster (Fig. 6A). We performed both fixed-effect (FFX) and random-effect (RFX) Bayesian model selections (Penny, Stephan, Mechelli, & Friston, 2004) to verify which model maximised explanatory accuracy and minimised model complexity. In the top-down model (Fig. 6A left), the triggering input entered the model through the IFG and ventral ATL due to their established significance for semantic cognition. Five modulatory parameters (thick red arrows) were specified to examine whether inter-node communication was altered by task: *(i)* the mutual influence between ventral ATL hub and IFG control centre, *(ii)* the mutual influence between ventral ATL hub and its nearby lateral ATL; and *(iii)* we assumed that the connectivity between IFG and occipital ‘colour-concept’ area (i.e., the PPI effect) is driven by top-down messages from the IFG to downstream visual cortex. The bottom-up model (Fig. 6B right) was otherwise identical to the top-down model but differed in two aspects: first, the input entered the model via the medial occipital percept node; second, the connectivity identified by PPI was driven by the lateral occipital ‘colour-concept’ node triggering the IFG.

Both the FFX and RFX Bayesian selection methods showed that the top-down model overwhelmingly outperformed the other model (Fig. 6A middle). We subsequently tested endogenous parameters (baseline strength of connection, without task-related modulation) of this winning top-down model and the results showed that all links were significantly excitatory (relative to zero, all *p*s < 1 × 10^−10^). Crucially, different task conditions significantly modulated effective connectivity (*F*_8, 136_ = 23.38, *p* < .001, *η*_p_^2^ = .57; see Fig. 6B). Closer inspection of the results revealed two distinct patterns of contextually-driven connectivity. Performing the associative-semantics task significantly strengthened the modulatory impact from the ventral ATL hub to IFG (simple main effect: *F*_2, 34_ = 30.85, *p* < .001) and from the hub to lateral ATL (*F*_2, 34_ = 40.39, *p* < .001), as well as the reciprocal effect from lateral ATL to the hub (*F*_2, 34_ = 12.29, *p* < .001). By contrast, the more effortful colour knowledge task relied on the IFG – performing this task significantly enhanced the impact that the IFG wielded on the occipital ‘colour-concept’ cluster, relative to other tasks (*F*_2, 34_ = 3.86, *p* = .03). The modulatory impacts that the IFG exerted on the ventral ATL hub did not differ between tasks (*F*_2, 34_ = 1.09, *p* = .34, *n.s.*), although a planned *t*-test revealed that the modulation is significantly greater than zero for the colour task (*p* = .02).

### 3.7 ROC analysis

**Fig. 7:**
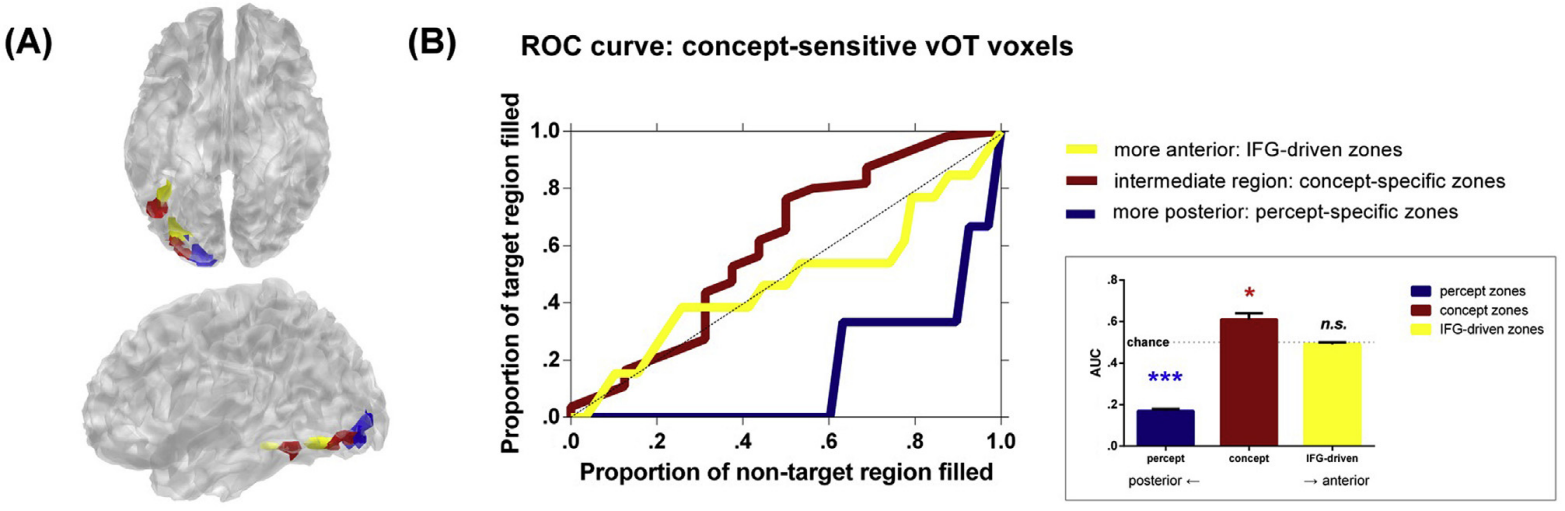
(A) Cortical layout of occipitotemporal voxels sensitive to hue perception (blue), colour knowledge (red), and communication with the IFG during retrieval of colour knowledge (yellow), thresholded at *p* < .005 (voxel intensity) and at least 270 mm^3^ (cluster size). **(B)** ROC analysis for the distribution of occipitotemporal voxels sensitive to colour concept (colour pairing > semantic pairing). Receiver operating characteristic curves, which show how each of the target zone is filled as an increasing number of voxels are included, starting from the ones most active to colour concept, plotted as a function of different target zones (see main text for details). The inset box shows the area under the curve (AUC) of each ROC curve. Error bars represent ±1 SEM. Asterisks represent statistically significant difference from chance level (.5); *p < .001, ***p < 10^−10^.

Within the ventral-posterior occipitotemporal cortices, we observed that voxels sensitive to colour perception (localiser: coloured > greyscale), colour concept (main tasks: colour knowledge > associative semantics), and showing co-variance with the IFG (PPI effect: colour knowledge > associative semantics) occupied neighbouring and partially overlapped cortical zones. When rendered on the cortex (Fig. 7A), the spatial layout of the three types of colour-related voxels exhibited an evident continuum: colour perception was coded in posterior sections, colour knowledge occupied intermediate zones, and concept-related communication with the IFG occurred in anterior patches of visual cortex. To characterise and quantify how the locations of the three types of colour processing were spatially organised in relation to each other, we performed a ROC analysis that has been exploited to tackle related issues and is able to circumvent the drawbacks of the conventional ROI approach (for precedent use of the ROC, see Konkle & Caramazza, 2013). The typical ROI analysis tests whether an independently defined ROI (e.g., colour-concept area) is engaged in a cognitive process of interest (e.g., hue perception or dialogue with the IFG), by averaging activation of all voxels within the ROI. This conventional approach fails to capture more nuanced variations in activity strength between voxels (due to the averaging process), and is validated using liberal statistical threshold (usually *α* = .05) to arbitrate if an effect is present within the ROI. By contrast, the ROC bypasses these issues by (*i*) considering the activation strength of each individual voxel and (*ii*) examining each voxel based on its percentile rank, covering the full breadth of intensity. We first identified all active voxels showing sensitivity to colour perception, colour concept and enhanced coupling with the IFG (i.e., the PPI effect of colour concept) in the left occipital lobe,^4^ threshold at *p* < .01. We deliberately adopted this liberal threshold to include voxels so as to gain insight about variation across the full breadth of activation level, from weakly to strongly responsive. All identified voxels were ranked based on their *t* value into a percentile (separately for the three types of colour processing). We then examined the location of each voxel, starting from the most to least active percentile of voxels, and computed the proportions of voxels that fell in a target zone (e.g., concept area) *vs*. non-target zones (e.g., percept and PPI areas). Note that this examination protocol was free from the constraints of spatial contiguity or anatomical location, enabling us to inspect which zone of the occipital cortex an individual active voxel was most/least likely to fall into. This procedure gave the ROC curves (with increasing numbers of voxels scrutinised, the curve plotting the proportion of target zone filled *vs*. non-target zone filled) and allowed computation of area under the curve (AUC). The ROC curves and AUC values were computed for each colour processing (concept, percept, and PPI) and each functionally-identified ROI as the target zone. If the ROC curve of a certain processing is situated well above/below the diagonal line (chance, AUC = .5), it means that the likelihood that activated voxels fall into the target zone is higher/lower than chance. If the ROC courses along the chance line with little deviation, it means that active voxels are equally likely to appear inside or outside this target zone.

Results of the ROC analysis complement the caudal-to-rostral gradient found in our ROI-vector analysis and further demonstrate the subtlety of representational transition. Most critically (see Fig. 7B), we found that voxels sensitive to colour concept (colour > semantic) were significantly more likely to fall into the intermediate concept-specific zone (AUC = .61; compared to chance, *Z* = 3.66, *p* < .001) and less likely to appear in the posterior percept-specific zone (AUC = .17; *Z* = 36.66, *p* < 1 × 10^−10^), with the likelihood of appearing in the most anterior IFG-driven zone equal to chance (AUC = .49; *Z* = 1.0, *p* = .32, *n.s.*). The AUC values of each curve also significantly differed from one another (all *Z*s > 3.79, *p*s < .0002; see Fig. 7B inset box). This pattern suggests a gradient structure at a micro within-occipital scale: Colour knowledge is represented in adjoining cortical zones anterior and lateral to those for colour percept. While a minimal number of voxels respond commonly to both concept and percept (as previous studies have demonstrated), the ROC showed that the great majority of activated voxels by concept tend *not* to fall into the perceptual zone.^5^ Furthermore, as illustrated in Fig. 7B, a proportion of concept-sensitive voxels appeared in the anterior zones where communication with the IFG occurred, sprinkled alongside the connectivity-sensitive voxels. The majority of concept-sensitive voxels are located in the intermediate area, sandwiched between percept- and IFG-driven zones. Together, the spatial layout of voxels constitutes a graded pattern, from sensory colour processing in the posterior section, through to conceptual colour processing that shifts anteriorly and laterally, to clusters that intensify connectivity with the IFG at the most anterior end of this gradation.

## 4 Discussion

We tested a key prediction of the CSC theory that the frontoparietal ‘control’ machinery regulates the hub-and-spoke ‘representation’ system depending on semantic contents and task characteristics. Specifically, a semantic task that requires pairing semantic concepts in an atypical/infrequent manner (or one that requires high degree of semantic specificity) would need greater executive control, constraining how neural signals propagate within the hub-and-spoke system. Compared to a typical associative-semantic task, we found that atypical pairing using specific canonical colour enhanced activity of the prefrontal and parietal ‘control’ regions, replicating previous work using similar designs to the present study (e.g., Thompson-Schill, D'Esposito, Aguirre, & Farah, 1997). The prefrontal activation might reflect its role in sustaining a task-defined attention-set and coordinating other brain regions to associate two ostensibly unrelated words using arbitrary rules requested by the task. Both tasks robustly activated the anterior sections of the FG, which has previously been proposed to be the centre–point of a graded representational ‘hub’ for semantic processing (Lambon Ralph et al., 2017). In addition, there was a gradient along the inferior temporal gyrus, with its caudal sector favouring the colour task (perhaps reflecting embodied colour simulation) and rostral sector preferring the typical association task. Furthermore, the PPI and DCM analyses revealed distinct patterns of connectivity for different tasks: Compared to the less effortful semantic task, PPI detected stronger coupling between the IFG (a key node of the ‘control’ network) and the occipitotemporal ‘spoke’ area (which adjoins the regions for colour perception, see below). DCM further revealed that this coupling reflects directional modulation from the IFG to the occipitotemporal cortex, implying strategic use of colour imagery during the task. Thus, while the two tasks engage largely overlapping prefrontal and ventral temporal cortices, the key distinction of their neural architecture lies in greater involvement of the frontoparietal control system during colour knowledge and the connectivity whereby the IFG attains embodied-visual information from the colour-related cortex. The absence of a direct IFG-ATL linkage in the PPI results is somewhat surprising – we speculate that this might be due to a lack of statistical power to detect the subtle PPI effect. Specifically, largely overlapping regions of the IFG and ATL were recruited by both the colour and semantic tasks. When the strong main effect of task-driven activation was partialled out, we might not have sufficient power to detect the more elusive PPI-related changes in connectivity (especially when the PPI-driven area might be overlapped with task-driven zones of ATL and IFG). All in all, our results highlight the complementary roles of the control and representation systems in supporting flexible use of semantic knowledge in a wide variety of contexts.

Our findings lend support to a key tenet of the CSC theory – semantic cognition is subserved by two functionally and anatomically distinct yet interacting machineries. First, a ‘hub-*and*-spoke’ system is in charge of representing *semantic content*: knowledge about perceptual features (e.g., ketchup is red) relies on modality-specific cortices (spoke) while a coherent concept (e.g., ketchup as fusion of multiple perceptual and functional traits) is built upon a ‘coalition’ of a polymodal hub (the anterior FG/vATL) and various modality-specific spokes (Pobric et al., 2010, Rogers et al., 2004). Second, an executively-related machinery for *semantic control* is implemented primarily by the left frontoparietal network (Badre et al., 2005, Jefferies and Lambon Ralph, 2006). This network constantly interacts with the hub-and-spokes structure to promote efficacious selection of task-relevant knowledge from a multitude of information in the representational database. These two neural systems can be selectively lesioned, causing *double dissociation*: Whereas ATL atrophy leads to semantic dementia (erosion of semantic memory with preserved capability of semantic control), frontoparietal stroke leads to semantic aphasia (inability to select appropriate pieces of semantic information, exacerbated by infrequent or unfamiliar meaning, while semantic memory *per se* is intact; for example, see Thompson et al., 2018, Thompson et al., 2017). It is noteworthy that we chose to focus on the IFG in the present study owing to its established role in selecting context-relevant semantic information, particularly under unfamiliar or ambiguous situations. Apart from the IFG, however, the intraparietal sulcus (IPS) and temporoparietal junction have been known to underpin semantic control (e.g., Davey et al., 2016, Whitney et al., 2011; for review, see; Noonan, Jefferies, Visser, & Lambon Ralph, 2013). In fact, our data also revealed these regions: in the GLM contrast between tasks (Fig. 2), the more demanding colour task induced greater activation of both the IFG and IPS; in the PPI results (Fig. 5), the colour task drove the IFG to become more connected with the IPS and a superior temporoparietal cluster. These are compatible with the literature and support the notion that regions other than the IFG are involved in semantic control. Together, there appears to be a triangulation (polymodal hub, unimodal spokes, and executive mechanism) of neurocomputation underling semantic cognition.

Our findings highlight the importance of connectivity in understanding semantic processing and the potential jeopardy of focusing solely on modality-specific regions, echoing recent proposals (Binney et al., 2012, Chen et al., 2017, Mahon, 2015a, Mahon, 2015b, Mahon, 2015c). Indeed, complete models of semantic cognition need to be able to surmount two challenges: *(i)* they need to expound how functionally distinct and often anatomically remote modules cooperate to engender semantic knowledge, transcending beyond the regional function of each component; and *(ii)* they also need to take the intrinsic wiring of neural tracts into consideration when explaining how cortical response and connectivity are couched in and constrained by neurophysiological infrastructure. Using a series of novel analyses to address these issues, we unravelled the functional interactions among constituent components of the semantic network. Furthermore, we revealed the representational gradients of cortical activity, both at an *intra*-lobar scale (within-occipital) and an *inter*-lobar scale (encompassing the entire VTC, spanning across the occipital and temporal lobes), and how neural connectivity alters its dynamics in response to different contexts.

Utilising PPI and DCM connectivity analyses, combined with ROI-vectors, we mapped the semantic networks of the brain. It is important to note that the present results fit closely with the pattern of intra- and inter-lobar white-matter connections in both human and non-human primates (Binney et al., 2012, Jung et al., 2016, Moran et al., 1987). The gradual transition of ROI-vectors, shifting anteriorly from perceptual to conceptual processing, meshes with the pattern of long-range fasciculi and short-range U-fibres of VTC. Across the caudal to rostral VTC, multiple long-range fasciculi (including inferior/middle longitudinal, uncinate fasciculi, and anterior commissure) all terminate in the anterior temporal region. Across medial to lateral VTC, multiple short-range fibres connect neighbouring gyri (Bajada et al., 2017, Binney et al., 2012, Papinutto et al., 2016). These short- and long-range connections provide the neural scaffolding, wherein information converges at the anterior temporal region and amalgamates both within and across sensory modalities (Binney et al., 2012). With this graded and converging structure, neural processing shifts gradually from perceptual computations for a single modality towards aggregate computations for poly-modalities and higher-level semantics. There are also inter-lobar connections, such as the inferior fronto-occipital fasciculus (IFOF) that links the IFG to occipitotemporal regions, as well as the uncinate fasciculus (UF) that links the IFG to ATL. The functional network that we identified in the present study fits with the tractography evidence, suggesting that the IFOF and UF might serves as conduits allowing the prefrontal cortex to exert top-down modulation on posterior regions (Almairac, Herbet, Moritz-Gasser, de Champfleur, & Duffau, 2015). An anatomical connectivity ‘triangle’ is completed by the inferior longitudinal fasciculus which directly connects the ATL with occipitotemporal and more posterior striate and extrastriate cortices (Binney et al., 2012).

The gradient within the visual cortex for ‘concept *vs*. percept’ dovetails with previous work that colour knowledge activates regions in proximity to regions for hue perception (Hsu et al., 2012, Rugg and Thompson-Schill, 2013, Simmons et al., 2007). Critically, while concept and percept occupy partially overlapping regions, concept tends to be more anterior and lateral. This pattern offers clues about the dissociation of patients and healthy individuals' data. Specifically, in healthy participants retrieval of colour knowledge induces occipitotemporal activity adjacent to regions of colour perception. However, in neurological patients there is double dissociation between perceptual and conceptual deficits: some patients lose their ability to perceive and recognise colours but retain the knowledge about canonical object colour when probed using imagery tasks (Shuren, Brott, Schefft, & Houston, 1996), whereas other patients lose colour knowledge while keeping normal colour perception (Miceli et al., 2001, Stasenko et al., 2013). This dissociation fits our ROC analysis for voxel distribution that, despite them being represented in adjoining regions, the core substrates of percept and concept might still be separable.

Our results speak directly to a spectrum of views on the nature of semantic representations, spanning from strong ‘embodied’ theories (concepts are rooted in sensorimotor activation, simulating original experiences) through to symbolic accounts which postulate that concepts reflect ‘amodal’ representations (Fodor, 1983, Mahon, 2015b, Meteyard et al., 2012). A key advantage of the ‘embodied’ approaches is that the source of information from which concepts can be learnt is apparent. However, this view struggles to tackle the fact that a conglomeration of perceptual features, by themselves, is *not* sufficient for creating coherent, generalisable concepts (Lambon Ralph et al., 2010), thus eliciting critiques (Dove, 2016, Leshinskaya and Caramazza, 2016). In light of the criticisms, treatments have proposed that the neural substrates of object knowledge entails both the perceptual cortex that codes embodied-experiential attributes, plus a polymodal region on which information from different perceptual modules converges (Lambon Ralph et al., 2017, Mahon, 2015c, Rogers et al., 2004). Echoing these proposals, our connectivity analyses provide clear evidence that the coordinated interplay among unimodal (spoke), polymodal (hub), and executive regions underpins the retrieval of task-relevant semantic representations. The involvement of visual cortices in colour knowledge concurs with previous studies (e.g., Simmons et al., 2007) and argues against a sharp separation between percept and concept. Critically though, the involvement of the polymodal ATL and the executive-related IFG, underlines the need for an additional contribution from a transmodal level so that the semantic system is able to establish accurate links between perceptual attributes (canonical colour) and their multimodal properties (object identity). These results also fit with accruing evidence that the ATL, particularly its ventral sector, serves the need to represent both concrete and abstract knowledge irrespective of input modality (Lambon Ralph et al., 2017, Peelen and Caramazza, 2012, Visser et al., 2011) and contributes directly to colour knowledge (Adlam et al., 2006, Chiou and Lambon Ralph, 2016, Chiou et al., 2013, Ikeda et al., 2006, Rogers et al., 2007), establishing the status of the ventral ATL as the centre–point of a graded, transmodal hub. As we demonstrated in the present work, the polymodal hub (ATL), unimodal spoke (occipitotemporal cortex in the case of colour knowledge), and executive system (IFG) work as a flexible, task-related coalition to represent different forms of semantic knowledge.

## Appendix A Supplementary data

mmc1

## Footnotes

1 It is important to note that the differential level of automaticity/difficulty between the colour and semantic task could be driven by two different (but not mutually exclusive) factors – (i) semantic precision: the colour task involves scrutiny of a very precise aspect of meaning (what colour is it?), whereas the semantic task involves broader, more miscellaneous types of associative semantics; (ii) typicality: pairing two concepts based on canonical colour is a less typical/familiar operation, compared to pairing based on associative meaning. Also note that while taxonomic category (ketchup with mustard) and semantic relationship (ostrich with eggshell) are understood as different conceptual constructs, they do *not* differ in neural representation but instead engage highly overlapping regions of the frontotemporal network, including the IFG and ATL (Jackson, Hoffman, Pobric, & Lambon Ralph, 2015). This indicates that while the different semantic targets might be associated with the probes in some subtly distinct ways, their neural underpinnings are largely common and such differences are unlikely be a confounding factor to our results.

2 The CSC theory maintains that both sides of the ATL ‘hubs’ contribute to semantic processing (Lambon Ralph et al., 2017). However, meta-analysis evidence (Rice, Hoffman, & Lambon Ralph, 2015) has shown that while both sides participate in semantic processing, there is a reliable activation asymmetry for certain stimulus types: the left ATL is disproportionately engaged by written words and speech production, whilst non-verbal (e.g., images) or spoken word materials generate bilateral activations. The meta-analysis results are consistent with the predominantly left-lateralised activation that we observed in the present study, due to our use of written word stimuli. Thus, we chose to focus on the left ATL due to its established robustness to survive stringent thresholds.

3 This relatively liberal threshold (*p* < .005, cluster size ≥ 270 mm^3^) was purposely used to detect the very subtle ‘embodied colour’ effect (i.e., colour knowledge > semantic association, testing its extent of overlap with the localiser results of coloured > greyscale) specifically in the ventral occipital and temporal cortices. We arrived at the threshold based on the simulation results by Lieberman and Cunningham (2009) that a whole-brain search thresholded using voxel intensity (*p* < .005) and cluster size (extending contiguously for 10 voxels/270 mm^3^) produced a desirable balance between Type I and II error rates, comparable to corrected multiple comparison using a false discovery rate (FDR) of *p* < .05 conventionally acceptable in behavioural literature. It is important to stress that, as far as we are aware, all previous studies had to use liberal thresholds to detect this type of embodied colour effects. For instance, Hsu, Kraemer, et al. (2011) did not apply any pre-determined threshold, and instead selected the top 100 maximally-responsive voxels that were active for hue perception to test the embodied colour effect. Similarly, Hsu, Frankland, and Thompson-Schill (2012) also used a threshold-free approach, selecting the top 50 hue-sensitive voxels to test the embodied effect within the fusiform and lingual gyri. Simmons et al. (2007) used *p* < .0001 for voxel intensity and 135 mm^3^ for cluster size (which is equivalent to only 5 voxels). All these studies found that the embodied effect did not survive whole-brain interrogation with more stringent thresholds, and minimal overlap was found between hue perception and colour knowledge (e.g., Hsu, Kraemer, et al. (2011) observed only 10 voxels overlapped in the left fusiform and 2 voxels in the left lingual gyrus). All studies had to use ROI analysis to detect the embodied effect. In the main text of Results section 3.3, we discuss the subtlety of this embodied colour effect, and the need to consider the broad neural architecture outside the occipitotemporal ROIs of hue perception when studying the neural basis of colour knowledge.

4 Note that the PPI results showed that the IFG was more connected with occipitotemporal cluster only in the left hemisphere. Hence, when conducting the ROC analysis we focused on the left occipitotemporal cortex to study the locations of PPI clusters in relation to areas of hue perception and colour knowledge.

5 We performed the same analysis on the occipital voxels sensitive to colour percept (coloured > greyscale). Results showed that voxels sensitive to hue perception were overwhelmingly *more* likely to fall into the posterior percept-specific zone (AUC = .99; compared to chance, *Z* = 24.7) and significantly *less* likely to fall into either the intermediate concept-specific zone (AUC = .28: *Z* = 110.5) or the most anterior PPI-defined zone (AUC = .22: *Z* = 28). Comparison of the latter two zones revealed that percept-voxels were more likely to fall in the concept zone than PPI zone (*Z* = 6.52, all *p*s < .001). Similar to patterns of the concept-voxels, the percept-voxels appear distributed in a graded fashion, with the majority of them congregating in the posterior occipital areas, some appearing in the nearby ‘colour-concept’ areas, and a tiny proportion appearing in the most anterior PPI-defined region.
