# Supplementary material for: Controlled semantic cognition relies upon dynamic and flexible interactions between the executive ‘semantic control’ and hub-and-spoke ‘semantic representation’ systems
Source: Cortex. 2018 Jun;103:100–16. doi: 10.1016/j.cortex.2018.02.018 (PMC6006425; doi:10.1016/j.cortex.2018.02.018)
Supplement: mmc1 [file mmc1.pdf]

# SUPPLEMENTAL INFORMATION

## Controlled semantic cognition relies upon a dynamic and flexible interaction between the executive 'semantic control' and hub-and-spoke 'semantic representation' systems

Rocco Chiou, Gina F. Humphreys, JeYoung Jung, & Matthew A. Lambon Ralph

Neuroscience and Aphasia Research Unit, Division of Neuroscience and Experimental Psychology, School of Biological Sciences, University of Manchester, United Kingdom

### CONTENTS

***A.*** Materials presented in the colour knowledge task:
- I. Table A (p. 2)
- II. Table legend (p. 2)

***B.*** Materials presented in the semantic association task:
- I. Table B (p. 3)
- II. Table legend (p. 3)

***C.*** Example stimuli presented in the visual matching control task:
- I. Supplemental figure 1 (*Fig. S1,* p. 4)
- II. Figure legend (p. 4)

***D.*** Supplemental ROI analysis of the default node network (DMN)
- I. Supplemental figure 2 (*Fig. S2,* p. 5)
- II. Figure legend (p. 5)

## A. Materials presented in the task of arbitrary pairing by canonical colour

| *PROBE* | *TARGET* | *FOIL* | *PROBE* | *TARGET* | *FOIL* |
|---|---|---|---|---|---|
| cork | chocolate | elephant | pencil lead | raven | grapefruit |
| ocean | jeans | spaghetti | ballet shoes | piglet | aubergine |
| pumpkin | clownfish | concrete | tennis ball | kiwifruit | coffee |
| spades (cards) | night sky | tonic water | barrel | calzone | popcorn |
| deer | viola | lawn | celery | moss | cannon |
| post box | cranberry | tyre | pie crust | oak wood | lake |
| pea | jade | yacht | robin breast | fire engine | grape juice |
| crown | omelette | clover | gorilla | piano | frost |
| cherry | fire extinguisher | saxophone | courgette | praying mantis | fortress |
| flour | lotion | rhubarb | violin | almond | dolphin |
| peanut butter | cardboard | cherry blossom | egg yolk | mango | vineyard |
| dough | iceberg | shamrock | baseball mitt | gravy | diamond |
| Latte | chestnut | quartz | staple | scalpel | autumn leaves |
| mistletoe | emerald | tuxedo | timber | saddle | sapphire |
| blusher | radish | vapour | toffee | whiskey | ambulance |
| wood veneer | camel | coriander | hot chocolate | moccasin | baseball |
| glacier | yoghurt | Sphinx | dove | cloud | snail |
| peacock tail | wreath | zebra | flamingo | lipstick | artichoke |
| algae | wasabi | Tarmac | gingerbread | cigar | rhino |
| granny smith apples | grass | sea | mint ice cream | fern | nappy |
| igloo | rice | seaweed | ivy | leek | petroleum |
| telephone box | raspberry | canvas | garlic | lamb | ginger hair |
| bread | chipmunk | peppermint | walnut | doe | Sprite |
| bronze medal | pine cone | glue | dirt | bran | beetroot |
| teeth | whipped cream | avocado | basil | meadow | stainless steel |
| rubber duck | lemon | hedgehog | ant | date | pickles |
| spinach | football pitch | heart | iguana | lime | chessboard |
| caterpillar | asparagus | turkey | pool table | Brussels sprout | sour cream |
| broccoli | frog | antler | ladybird | paprika | tunnel |
| golf ball | ivory | earthworm | hay | faeces | thermometer |
| mustard | smiley | hawk | denim | sky | sausage |
| London bus | rose | wasp | basketball | tangerine | turnip |
| pepperoni | brick | syringe | tofu | cotton wool | bruise |
| cauliflower | lab coat | cockroach | strawberry | stop sign | Statue of Liberty |
| marmalade | goldfish | soil | blood | chilli | pyramid |
| floorboard | beer | cabbage | wheat grain | Espresso | teardrop |
| tomato | traffic cone | TARDIS | meatball | brunette hair | thunder |
| canary | banana | cobweb | Tippex | snow | antelope |
| chips | daffodil | crow | duckling | dandelion | plum |
| bridal dress | polar bear | kangaroo | cactus | grasshopper | Yorkshire pudding |
| toilet paper | mashed potato | prune | rash | smoked salmon | spanner |
| ostrich | sea lion | cheese | sheep | napkins | sweet potato |
| milk | snowman | rust | ruby | pomegranate | hornet |
| coal | chimpanzee | lagoon | poppy | wound | Pepsi |
| Fanta | carrot | pearl | lavender | amethyst | ointment |

***Table A.*** Materials in the colour knowledge task; in each display of triplet stimuli, the probe item was shown above the centre; the target and foil were situated below, equally likely to be on the left or right.

# B. Materials presented in the task of semantic pairing by usual meaning

| *PROBE* | *TARGET* | *FOIL* | *PROBE* | *TARGET* | *FOIL* |
|---|---|---|---|---|---|
| cork | wine bottle | elephant | pencil lead | rubber | grapefruit |
| ocean | scuba diving | spaghetti | ballet shoes | Swan Lake | aubergine |
| pumpkin | Halloween | concrete | tennis ball | racket | coffee |
| spades (cards) | poker games | tonic water | barrel | brewery | popcorn |
| deer | hunter | lawn | celery | blender | cannon |
| post box | postman | tyre | pie crust | recipe | lake |
| pea | risotto | yacht | robin breast | nest | grape juice |
| crown | emperor | clover | gorilla | zoo | frost |
| cherry | crumble | saxophone | courgette | salad bowl | fortress |
| flour | pastry | rhubarb | violin | auditorium | dolphin |
| peanut butter | knife | cherry blossom | egg yolk | rooster | vineyard |
| dough | rolling pin | shamrock | baseball mitt | catcher | diamond |
| Latte | cafe | quartz | staple | ring binder | autumn leaves |
| mistletoe | Santa | tuxedo | timber | chainsaw | sapphire |
| blusher | powder case | vapour | toffee | condensed milk | ambulance |
| wood veneer | furniture | coriander | hot chocolate | milkshake | baseball |
| glacier | Norway | Sphinx | dove | pigeon | snail |
| peacock tail | feather | zebra | flamingo | wetland | artichoke |
| algae | microbes | Tarmac | gingerbread | bakery | rhino |
| granny smith apples | farmer | sea | mint ice cream | refrigerator | nappy |
| igloo | Eskimos | seaweed | ivy | wall | petroleum |
| telephone box | handset | canvas | garlic | chive | ginger hair |
| bread | toaster | peppermint | walnut | nutcracker | Sprite |
| bronze medal | athlete | glue | dirt | vacuum cleaner | beetroot |
| teeth | tongue | avocado | basil | plant pot | stainless steel |
| rubber duck | bathtub | hedgehog | ant | pesticide | pickles |
| spinach | nutrient | heart | iguana | reptile | chessboard |
| caterpillar | butterfly | turkey | pool table | billiards | sour cream |
| broccoli | pasta | antler | ladybird | garden | tunnel |
| golf ball | tee | earthworm | hay | baler | thermometer |
| mustard | ketchup | hawk | denim | textile | sausage |
| London bus | passenger | wasp | basketball | backboard | turnip |
| pepperoni | pizza | syringe | tofu | chopsticks | bruise |
| cauliflower | soup | cockroach | strawberry | smoothie | Statue of Liberty |
| marmalade | spoon | soil | blood | bandage | pyramid |
| floorboard | mop | cabbage | wheat grain | scarecrow | teardrop |
| tomato | hamburger | TARDIS | meatball | chilli sauce | thunder |
| canary | cage | cobweb | Tippex | typo | antelope |
| chips | frying pan | crow | duckling | goose | plum |
| bridal dress | groom | kangaroo | cactus | desert | Yorkshire pudding |
| toilet paper | diarrhoea | prune | rash | clinic | spanner |
| ostrich | eggshell | cheese | sheep | shepherd | sweet potato |
| milk | cereal | rust | ruby | necklace | hornet |
| coal | chimney | lagoon | poppy | war memorials | Pepsi |
| Fanta | Coke | pearl | lavender | air freshener | ointment |

***Table B.*** Materials in the semantic association task; in each display of triplet stimuli, the probe item was shown above the centre; the target and foil were situated below, equally likely to be on the left or right.

## C. Example stimuli presented in the control visual matching task

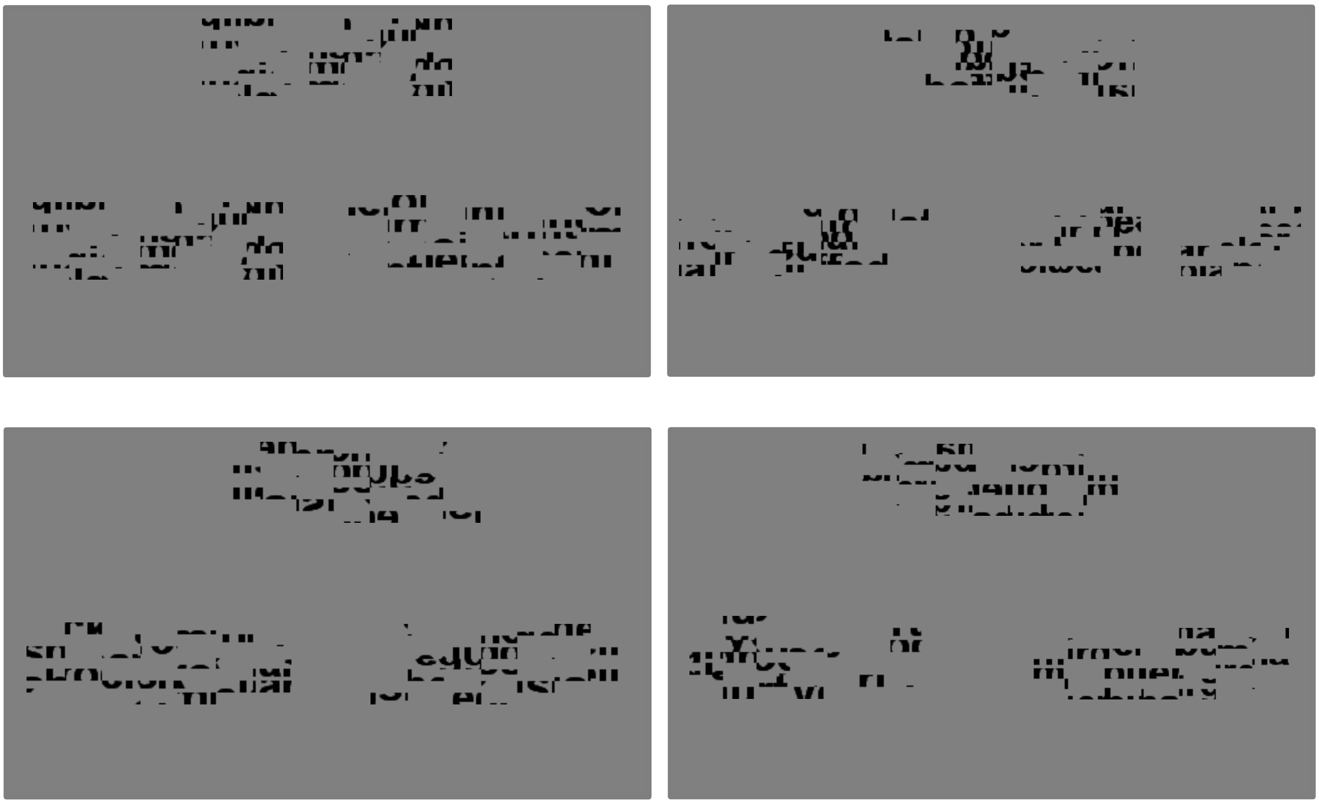

***Fig. S1*** Four example displays in the control visual matching task; in each trial, a triplet of scrambled pattern was shown; the top item was the probe; one of the bottom items was the left-right mirror inverse of the upper probe (target) and the other item was the foil; the targets and foils were equally likely to be on the left and right.

## D. Supplemental ROI analysis of the default mode network (DMN)

In one of the PPI analyses reported in the main article, we found that various regions of the default mode network showed stronger functional coupling with the left IFG when we set the seed region at the left IFG and searched for areas connected more strongly during the semantic association than colour knowledge task. This could result from two possible mechanisms: *(i)* the semantic task activated the DMN more than the colour task or *(ii)* the colour task deactivated the DMN more than the semantic task. To test these two alternatives, we carried out further ROI analysis to investigate, compared to rest, how the DMN reacted to the three main tasks. The tests were performed on three spherical ROIs (6-mm of radius) centred at peak activations of the DMN – the right medial-prefrontal cortices (mPFC), right angular gyrus (AG), and right precuneus. The analysis showed that, although all of the three tasks deactivated the DMN, the magnitude of deactivation was significantly stronger during the colour task than other conditions, indicating that the pattern of PPI results was driven by greater deactivation of DMN during the colour task.

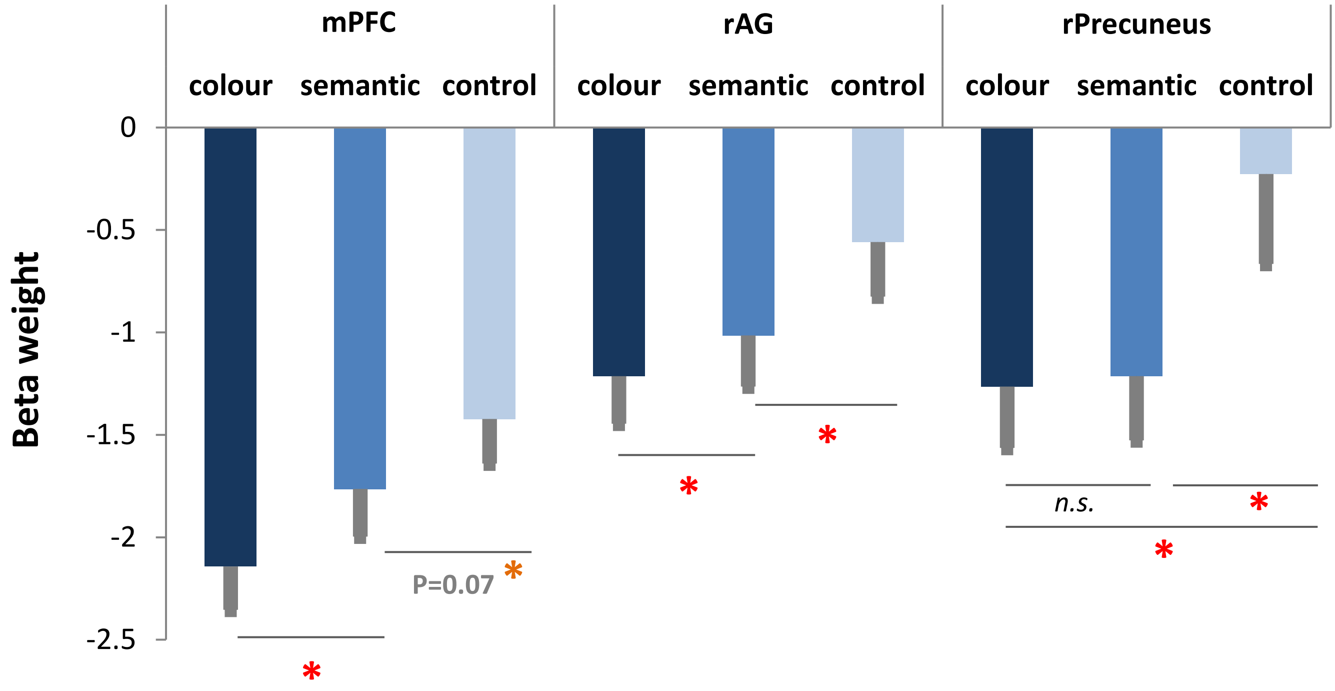


***Fig. S2*** Results of the supplemental ROI analysis, comparing each condition against rest. Error bars represent 1 SEM. Note that negative beta weights represent deactivation. Asterisks represent $p < 0.05$ for pair-wise comparison for the amount of deactivation between tasks (corrected for false discovery rate).
